# Sex Differences in Trends of Firearm Suicide Among Older Adults, 2014 to 2023

**DOI:** 10.1001/jamanetworkopen.2025.28709

**Published:** 2025-08-25

**Authors:** Ziming Xuan, William Y. Xuan, Mark S. Kaplan

**Affiliations:** 1 Department of Community Health Sciences, Boston University School of Public Health, Boston, Massachusetts; 2 Department of Epidemiology, Boston University School of Public Health, Boston, Massachusetts; 3 Brookline High School, Brookline, Massachusetts; 4 Luskin School of Public Affairs, University of California, Los Angeles

## Abstract

This cross-sectional study examines trends in suicide deaths by firearm among US adults aged 65 years or older between 2014 and 2023.

## Introduction

Firearms are the leading method of suicide among adults aged 65 years or older. Older men are 13 times more likely to die by firearm suicide compared with older women.^1^ However, female gun ownership has surged in recent years, with nearly one-half of all new gun owners being women.^2,3,4^ This shift highlights the need to examine trends of firearm suicides among older adults.

## Methods

In this cross-sectional study, we collected counts, crude rates, and age-adjusted rates of suicide deaths among older adults from the CDC WONDER database from January 1, 2014, through December 31, 2023. As data are deidentified and publicly available, local ethics review and informed consent were not required in accordance with the Common Rule. This study followed the STROBE reporting guideline.

We calculated the ratio of firearm suicides to all suicides (FS/S) for each year and conducted linear regression models to examine rate trends and FS/S ratios over the study period. We also calculated a sex-by-trend interaction term to determine whether differential trends existed and examined state-level associations in FS/S ratios between men and women using Pearson correlation (eMethods in Supplement 1). As the overwhelming majority of suicide deaths in the dataset were among older White adults (96.7%), no analysis was performed regarding race and ethnicity. Data were analyzed using SAS, version 9.4 (SAS Institute Inc). A 2-sided *P* < .05 was considered significant.

## Results

A total of 63 559 older individuals who died by firearm suicide were included (median [IQR] age, 75 [69-81] years; 8.8% female and 91.2% male). Among women, the FS/S ratio increased from 34.9% (456 of 1306 deaths) to 38.5% (714 of 1855 deaths) (β = 0.42; 95% CI, 0.25-0.58) (Table 1). The crude firearm suicide rates per 100 000 population increased from 1.76 in 2014 to 2.20 in 2023 (β = 0.04; 95% CI, 0.02-0.06). In contrast, although men had substantially higher firearm suicide rates, their FS/S ratios remained unchanged over time (β = 0.07; 95% CI, −0.04 to 0.19). The interaction between sex and trend increased significantly among women compared with men (*P* = .005).

**Table 1.** US Suicide Rates and FS/S Ratios, 2014 to 2023

Cohort	Year										Trend test, β (95% CI)^a^	*P* value
	2014	2015	2016	2017	2018	2019	2020	2021	2022	2023		
**Women aged ≥65 years**												
Firearm suicides, No.	456	472	526	505	558	563	552	593	643	714	NA	
Rate, per 100 000 population	1.76	1.77	1.92	1.78	1.92	1.88	1.79	1.94	2.02	2.20	0.04 (0.02 to 0.06)	.01
All suicides, No.	1306	1369	1450	1483	1526	1553	1494	1565	1723	1855	NA	
Rate, per 100 000 population	5.04	5.13	5.28	5.24	5.24	5.18	4.84	5.11	5.41	5.71	0.04 (−0.01 to 0.08)	.17
FS/S ratio, %	34.9	34.5	36.3	34.1	36.6	36.3	36.9	37.9	37.3	38.5	0.42 (0.25 to 0.58)	.01
**Men aged ≥65 years**												
Firearm suicides, No.	4920	5039	5230	5491	5817	5829	5920	6342	6724	6665	NA	
Rate, per 100 000 population	24.18	23.89	24.00	24.33	24.96	24.21	23.85	25.15	25.93	24.89	0.15 (0.03 to 0.26)	.04
All suicides, No.	6396	6543	6754	7085	7576	7620	7643	8087	8715	8582	NA	
Rate, per 100 000 population	31.43	31.02	30.99	31.40	32.51	31.65	30.79	32.07	33.61	32.04	0.16 (−0.01 to 0.32)	.09
FS/S ratio, %	76.9	77.0	77.4	77.5	76.8	76.5	77.5	78.4	77.2	77.7	0.07 (−0.04 to 0.19)	.24

Abbreviations: FS/S, firearm suicide to total suicide; NA, not applicable.

^a^ The β-coefficient in the linear trend test regression model corresponded to an annual change of the crude rate.

The FS/S ratios of men and women across states were significantly correlated (*r* = 0.88; *P* = .01), exhibiting substantial variation among states (Table 2). Alabama and Mississippi had the highest FS/S ratios for older men (92.7% and 91.3%, respectively), as well as for older women (68.1% and 68.0%, respectively), surpassing the FS/S ratios for older men in Massachusetts (41.6%) and New York (52.4%).

**Table 2.** Aggregated State Firearm Suicide Rates and FS/S Ratios, 2014 to 2023

State	Firearm suicide rate, per 100 000 population^a^		FS/S ratio, %^b^	
	Men	Women	Men	Women
Alabama	34.45	3.32	92.7	68.1
Alaska	34.72	Suppressed	84.9	Suppressed
Arizona	37.63	3.63	82.8	42.7
Arkansas	35.98	3.23	89.7	59.9
California	19.49	1.27	65.9	22.5
Colorado	33.24	2.88	78.1	33.2
Connecticut	12.70	Suppressed	55.3	Suppressed
Delaware	19.79	Suppressed	75.6	Suppressed
District of Columbia	Suppressed	Suppressed	Suppressed	Suppressed
Florida	28.17	2.41	76.1	35.7
Georgia	29.05	3.20	87.1	59.7
Hawaii	8.60		39.1	
Idaho	39.06	3.07	85.0	44.7
Illinois	16.49	1.05	68.4	27.3
Indiana	28.52	1.53	83.4	45.0
Iowa	23.61	0.87	78.8	24.3
Kansas	29.01	1.61	84.4	33.3
Kentucky	35.17	2.93	87.0	62.6
Louisiana	28.78	3.02	88.8	65.8
Maine	30.34	Suppressed	77.8	Suppressed
Maryland	17.68	1.03	71.2	24.7
Massachusetts	7.09	Suppressed	41.6	Suppressed
Michigan	22.73	1.49	77.8	33.0
Minnesota	17.21	0.86	71.7	20.7
Mississippi	32.35	3.25	91.3	68.0
Missouri	32.08	2.71	85.9	50.3
Montana	46.14	2.67	82.9	41.2
Nebraska	19.92	Suppressed	78.8	Suppressed
Nevada	43.24	4.96	79.5	46.8
New Hampshire	23.52	1.86	71.1	28.4
New Jersey	9.18	0.52	52.8	14.6
New Mexico	40.09	4.44	82.6	50.9
New York	9.22	0.31	52.4	8.7
North Carolina	27.10	2.59	84.2	47.4
North Dakota	22.53	Suppressed	85.4	Suppressed
Ohio	25.49	1.50	81.8	35.8
Oklahoma	38.03	3.19	87.6	57.1
Oregon	36.32	3.77	78.6	38.5
Pennsylvania	24.38	1.33	76.8	31.3
Rhode Island	8.35	Suppressed	46.9	Suppressed
South Carolina	27.82	2.74	85.9	49.5
South Dakota	22.98	Suppressed	81.5	Suppressed
Tennessee	34.73	3.23	87.9	53.6
Texas	26.42	2.45	84.0	52.1
Utah	29.42	1.90	81.4	25.7
Vermont	30.07	Suppressed	77.8	Suppressed
Virginia	26.50	2.46	82.0	44.3
Washington	27.35	2.16	74.7	29.7
West Virginia	38.98	3.29	89.7	61.5
Wisconsin	21.31	1.15	75.3	25.4
Wyoming	49.09	4.60	86.4	48.9

Abbreviation: FS/S, firearm suicide to total suicide.

^a^ Pearson *r* = 0.89; *P* = .01.

^b^ Pearson *r* = 0.88; *P* = .01.

## Discussion

This cross-sectional study reveals an increasing trend of suicide deaths by firearm among older women. Although the firearm suicide rate and FS/S ratio among older men remain high, the trend among older women may have important long-term implications. The proportion of older adults in the US is projected to grow from 17.3% in 2022 to 21.6% by 2040, with women accounting for a larger share of this increase.^5^ Research has shown a steady decline in the FS/S ratio among women from 41.5% in 1991 to 35.3% in 2013.^6^ However, our analysis of data from 2014 to 2023 highlights a significant upward trend among older women, suggesting a narrowing sex gap in firearm suicide that aligns with changes in gun ownership demographics.^3^ There was considerable variation across states. Many southern states exhibited high FS/S ratios for both sexes, which may be influenced by high rates of rural residency and permissive firearm policies.^1^ Key limitations included the short study period and lack of detailed demographic data, which may have led to small sample sizes and residual confounding (eg, poverty rate, percentage of college graduates).

Firearms were involved in nearly 40% of suicides among older women and 75% of suicides among older men during 2014 to 2023. It is essential to enhance access to mental health care for older adults, particularly in states with less restrictive firearm control policies and high levels of firearm ownership.

## Notes

Supplement 1.
eMethods.

Supplement 2.
Data Sharing Statement
