# Supplementary material for: Sex Differences in Trends of Firearm Suicide Among Older Adults, 2014 to 2023
Source: JAMA Netw Open. 2025 Aug 25;8(8):e2528709. doi: 10.1001/jamanetworkopen.2025.28709 (PMC12379079; doi:10.1001/jamanetworkopen.2025.28709)
Supplement: Supplement 1. — eMethods. [file jamanetwopen-e2528709-s001.pdf]

Supplemental Online Content

Xuan Z, Xuan WY, Kaplan MS. Sex differences in trends of firearm suicide among older adults, 2014 to 2023. *JAMA Netw Open*. 2025;8(8):2528709.
doi:10.1001/jamanetworkopen.2025.28709

eMethods

This supplemental material has been provided by the authors to give readers additional information about their work.

© 2025 Xuan Z et al. *JAMA Network Open*.

eMethods

We collected suicide counts, crude rates, and age-adjusted rates among older adults from the CDC WONDER database for the years 2014 to 2023. We calculated the firearm suicides to all suicides (FS/S) ratio for each year. We conducted separate linear regression models to examine the trends of these rates and FS/S ratios outcome over the study period. To evaluate the trend, we coded the year of 2014 as 1 and up to 10 for the year of 2023. Therefore, the beta coefficient in the linear trend test regression model corresponded to the annual change of the outcome. Additionally, to determine differential trends by sex, we constructed a linear model that included sex term (female vs. male), trend, and sex-by-trend interaction term. To examine geographic distribution of firearm suicide by states, we aggregated data for the study period and examined state-level association between men and women FS/S ratios by Pearson correlation. Our study followed the STROBE guideline. The analysis was conducted using SAS software (version 9.4) between February 11 and June 7, 2025. The statistical significance was determined by 95% confidence intervals and p-value less than 0.05.

© 2025 Xuan Z et al. *JAMA Network Open*.
