# Supplementary material for: Sex Differences in Trends of Firearm Suicide Among Older Adults, 2014 to 2023
Source: JAMA Netw Open. 2025 Aug 25;8(8):e2528709. doi: 10.1001/jamanetworkopen.2025.28709 (PMC12379079; doi:10.1001/jamanetworkopen.2025.28709)
Supplement: Supplement 2. — Data Sharing Statement [file jamanetwopen-e2528709-s002.pdf]

Data Sharing Statement

Xuan. Sex Differences in Trends of Firearm Suicide Among Older Adults, 2014 to 2023. *JAMA Netw Open*. Published August 25, 2025. doi:10.1001/jamanetworkopen.2025.28709

Data
Data available: Yes
Data types: Data (not involving human participants)
How to access data: Data can be requested from Dr. Ziming Xuan at zxuan@bu.edu
When available: With publication

Supporting Documents
Document types: Statistical/analytic code
How to access documents: Statistical/analytic code will be available from Dr. Ziming Xuan at zxuan@bu.edu
When available: With publication

Additional Information
Who can access the data: Anyone requesting the data
Types of analyses: All analyses involved in the article will be available.
Mechanisms of data availability: The data will be made with clear instructions therefore analyses can be completed with the provided code without investigator support.
